# Amniotic-fluid Lactoferrin: A Marker for Subclinical Intraamniotic Infection Prior to 32 Weeks Gestation

**DOI:** 10.1155/S1064744995000573

**Published:** 1995

**Authors:** Kimberly A. Heller, Phillip C. Greig, R. Phillip Heine

**Affiliations:** 1 Department of Obstetrics, Gynecology, and Reproductive Sciences University of Pittsburgh Pittsburgh PA USA; 2 Department of Obstetrics and Gynecology Duke University Durham NC USA; 3 Department of Obstetrics, Gynecology, and Reproductive Sciences Magee-Womens Hospital 300 Halket Street Pittsburgh PA 15213 USA

## Abstract

*Objective:* Lactoferrin is a glycoprotein released from the secondary granules of activated neutrophils in the setting of infection. The purpose of this study was to determine if amniotic-fluid (AF) lactoferrin levels are elevated in preterm labor (PTL) patients with subclinical intraamniotic infection (IAI).

*Methods:* AF samples were obtained from 186 pregnant patients with the following characteristics: group 1 - term, no labor; group 2 - preterm, no labor; group 3 - PTL with IAI; group 4 - PTL without IAI. Lactoferrin levels were measured with an enzyme-linked immunosorbent assay (ELISA).

*Results:* AF lactoferrin levels were elevated in normal gestation after 31 weeks (P < 0.0001). Lactoferrin levels were also higher in infected PTL patients compared with noninfected PTL patients at gestations ≤31 weeks (P = 0.005). An AF lactoferrin level of >2.5 μg/ml is highly suggestive of infection in PTL patients at <32 weeks, with an overall sensitivity of 82% and a specificity of 83%, when infection is defined as a positive AF culture or positive placental histology.

*Conclusions:* AF lactoferrin levels increase after 31 weeks in normal gestations, but lactoferrin levels >2.5 μg/ml in PTL patients before this gestational age are highly suggestive of IAI. AF lactoferrin levels may be a useful clinical tool for selecting those PTL patients who might benefit from antimicrobial therapy, closer observation, or early delivery.


Infectious Diseases in Obstetrics and Gynecology 3:179–183 (1995)
© 1996 Wiley-Liss, Inc.

### Amniotic-Fluid Lactoferrin: A Marker for Subclinical Intraamniotic Infection Prior to 32 Weeks Gestation

**Kimberly A. Heller, Phillip C. Greig, and R. Phillip Heine**

*Department of Obstetrics, Gynecology, and Reproductive Sciences, University of Pittsburgh, Pittsburgh, PA (K.A.H., R.P.H.); Department of Obstetrics and Gynecology, Duke University, Durham, NC (P.C.G.)*

Abstract

***Objective:*** Lactoferrin is a glycoprotein released from the secondary granules of activated neutrophils in the setting of infection. The purpose of this study was to determine if amniotic-fluid (AF) lactoferrin levels are elevated in preterm labor (PTL) patients with subclinical intraamniotic infection (IAI).

***Methods:*** AF samples were obtained from 186 pregnant patients with the following characteristics: group 1 - term, no labor; group 2 - preterm, no labor; group 3 - PTL with IAI; group 4 - PTL without IAI. Lactoferrin levels were measured with an enzyme-linked immunosorbent assay (ELISA).

***Results:*** AF lactoferrin levels were elevated in normal gestation after 31 weeks ($P < 0.0001$). Lactoferrin levels were also higher in infected PTL patients compared with noninfected PTL patients at gestations ≤31 weeks ($P = 0.005$). An AF lactoferrin level of >2.5 μg/ml is highly suggestive of infection in PTL patients at <32 weeks, with an overall sensitivity of 82% and a specificity of 83%, when infection is defined as a positive AF culture or positive placental histology.

***Conclusions:*** AF lactoferrin levels increase after 31 weeks in normal gestations, but lactoferrin levels >2.5 μg/ml in PTL patients before this gestational age are highly suggestive of IAI. AF lactoferrin levels may be a useful clinical tool for selecting those PTL patients who might benefit from antimicrobial therapy, closer observation, or early delivery. © 1996 Wiley-Liss, Inc.

Key words

Preterm labor, chorioamnionitis, decidual activation

Preterm labor (PTL) and delivery are significant causes of perinatal mortality and morbidity. In about 40% of cases, the underlying cause of PTL is unclear, particularly in the setting of PTL with intact membranes.[1] Mounting evidence suggests an infectious etiology for PTL by linking positive amniotic-fluid (AF) cultures, elevated AF cytokine levels, or histologic chorioamnionitis to PTL with intact membranes.[2-10] It is theorized that bacterial invasion of the decidua, membranes, or AF stimulates a maternal immune response that involves the release of cytokines along with neutrophil activation. This immune response, in turn, initiates prostaglandin production and a cascade of events leading to PTL.

Lactoferrin is an iron-binding glycoprotein present in a number of body fluids including breast milk, sweat, tears, and plasma.[11] It has also been reported to be present at low levels in AF in early pregnancy, with increasing levels in the third trimester. The source of increasing lactoferrin levels in the third trimester is thought to be decidua.[12] Lactoferrin is also known to be released from the secondary granules of activated neutrophils in the presence of inflammation.[11]

We hypothesized that AF lactoferrin levels

Address correspondence/reprint requests to Dr. Kimberly A. Heller, Department of Obstetrics, Gynecology, and Reproductive Sciences, Magee-Womens Hospital, 300 Halket Street, Pittsburgh, PA 15213.

Received December 16, 1995
Accepted January 24, 1996

would be elevated in idiopathic PTL secondary to decidual activation and that lactoferrin levels would be even more elevated in PTL due to intraamniotic infection (IAI).

### SUBJECTS AND METHODS

AF samples were obtained from 186 pregnant patients with intact membranes and no clinical-evidence of chorioamnionitis. Approval for this investigation was obtained from the appropriate institutional review board, and written informed consent was obtained from each patient. All specimens were obtained by transabdominal amniocentesis under continuous ultrasound guidance. The AF was transported to the laboratory in capped plastic syringes. A portion of the fluid was sent to the research laboratory for microbiologic evaluation. AF cultures were performed for aerobic and anaerobic bacteria, mycoplasmas, *Neisseria gonorrhoeae*, and *Chlamydia trachomatis*, as previously described.[13] The remaining fluid was immediately centrifuged at 500–800 *g* for 10 min and the supernatant was stored at −70°C for later evaluation of lactoferrin levels.

Placentas were collected from all patients who delivered within 72 h of amniocentesis and processed as previously described.[13] Histologic chorioamnionitis was defined by the criteria of Salafia et al.[14] The presence of grade 1 inflammation (inflammatory cell invasion of at least 5 PMNs into the amnion, chorion, subchorionic fibrin, or inner third of the umbilical-vein wall) or greater in any of 3 placental sections examined was used to define histologic chorioamnionitis. All specimens were examined by the same pathologist who was blinded to the patient's clinical course.

The AF samples previously stored at −70°C were thawed in batches, and the lactoferrin levels were determined with an enzyme-linked immunosorbent assay kit specific for lactoferrin (LEUKO-ELISA, Techlab, Blacksburg, VA). This assay has a detection limit of 4 ng/ml. The samples with lactoferrin levels above the upper limit of the assay's detection range were diluted and reassayed. The interassay and intraassay coefficients of variation were <10%. Lactoferrin assays were performed in the research laboratory of Magee-Womens Research Institute.

The patients were stratified into the following 4 groups based on clinical presentation:

Group 1: Term, no labor. Patients at ≥37 weeks, not in labor, undergoing amniocentesis for fetal lung maturity studies prior to scheduled repeat cesarean deliveries. N = 50.

Group 2: Preterm, no labor. Patients at 24–36 weeks gestation, not in labor, who were undergoing amniocentesis for genetic evaluation or AF bilirubin studies. A patient was excluded if the karyotype was abnormal or the fetus was subsequently found to be affected by maternal isoimmunization. N = 52. An additional 29 samples were collected from similar patients not in labor at 14–23 weeks gestation. Data from these samples were excluded from analysis with group 2, but were used in the analysis of lactoferrin levels vs. gestational age.

Group 3: PTL with IAI. Patients in PTL at 24–34 weeks gestation with intact membranes and subclinical IAI. Subclinical IAI was defined as a positive AF culture or placental histologic chorioamnionitis without clinical signs of infection. N = 20.

Group 4: PTL without IAI. Patients in PTL at 24–34 weeks gestation with intact membranes and without subclinical IAI. These patients had negative AF cultures and negative placental histology (N = 2) or negative AF cultures and no delivery within 72 h of the amniocentesis (N = 33). Total N = 35.

The exclusion criteria for all groups included multiple gestation, evidence of rupture of the membranes, diabetes, treatment with antibiotics within the previous 7 days, the presence of any condition requiring antimicrobial treatment, and abnormal karyotype. Further exclusion criteria for groups 3 and 4 included gestational age of <24 weeks or >34 weeks or cervical dilation >4 cm. Patients with clinical evidence of chorioamnionitis as defined by Gibbs et al.[15] were also excluded. PTL was defined as the presence of regular uterine contractions with a frequency of at least 10 contractions per hour and observed cervical change.

Analysis of the data was performed on the statistical program SYSTAT 5.2 for the Macintosh (SYSTAT, Inc., Evanston, IL). The Mann-Whitney U-test was used for nonparametric comparison of AF lactoferrin concentrations of the various groups. The Pearson correlation coefficient was used to evaluate the correlation between AF lactoferrin levels and gestational age. Statistical significance was defined as $P < 0.05$.

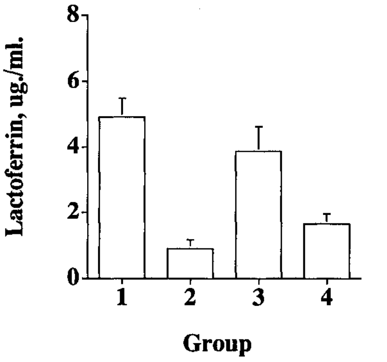

Fig. 1. Median lactoferin levels ± standard error by patient group. Group 1: Term, 37–42 weeks gestation, no labor. Group 2: Preterm, 24–36 weeks gestation, no labor. Group 3: Preterm labor, 24–34 weeks gestation, with IAI. Group 4: Preterm labor, 24–34 weeks gestation, without IAI. Group 1 vs. group 2, $P < 0.0001$; group 3 vs. group 4, $P = 0.057$.

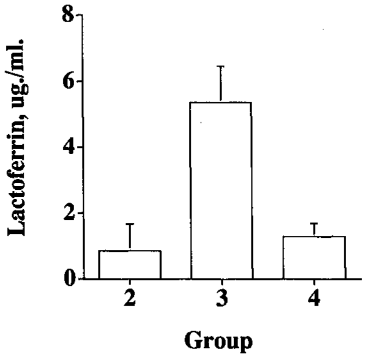

Fig. 3. Median lactoferrin levels ± standard error in all preterm patients at ≤31 weeks gestation. Group 2: Preterm, 14–31 weeks gestation, no labor. Group 3: Preterm labor, 24–31 weeks gestation, with IAI. Group 4: Preterm labor, 24–31 weeks gestation, without IAI. Group 2 vs. groups 3 and 4, $P = 0.03$; group 3 vs. group 4, $P = 0.005$.

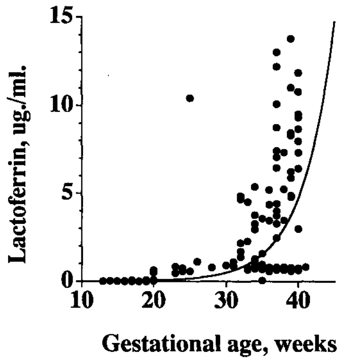

Fig. 2. Lactoferrin levels vs. gestational age in all nonlaboring patients. Significant relationship between lactoferrin level and gestational age by Pearson correlation, $P < 0.001$.

### RESULTS

The median lactoferrin levels in each group are shown in Figure 1. The median level of lactoferrin was low in nonlaboring preterm patients (group 2) and significantly higher in nonlaboring patients at term (group 1) ($P < 0.0001$). In the preterm patients, the median lactoferrin levels in the PTL groups (groups 3 and 4) were significantly higher than in nonlaboring preterm (PTL) patients (group 2) ($P = 0.002$).

The effect of gestational age on lactoferrin levels in nonlaboring patients is shown in Figure 2. Lactoferrin levels are quite low prior to 32 weeks gestation and rise markedly from 32 weeks to term. When we analyzed the data by the Pearson correlation, a significant relationship between gestational age and lactoferrin level was shown for nonlaboring patients ($P < 0.001$). Because of the gestational age effect illustrated in Figure 2, we analyzed the PTL data at gestational ages of <32 weeks separately, as shown in Figure 3. The median lactoferrin level in the infected PTL group (group 3) was significantly higher than in the noninfected PTL group (group 4) ($P = 0.011$), and both PTL groups had significantly higher lactoferrin levels than the preterm, no labor group (group 2) ($P = 0.03$).

The results of cultures, histology, and lactoferrin levels for the infected PTL group at ≤31 weeks gestation are shown in Table 1. In the PTL group at <32 weeks gestation, a lactoferrin level of >2.5 μg/ml had a sensitivity of 100% for the identification of a positive AF culture, with a specificity of 67%, a positive predictive value (PPV) of 46%, and a negative predictive value (NPV) of 100%. This same lactoferrin level of >2.5 μg/ml had a sensitivity of 82% for identification of positive histology, with a specificity of 83%, a PPV of 82%, and a NPV of 83%.

### DISCUSSION

These data suggest that AF lactoferrin levels in nonlaboring patients tend to be low, <2.5 μg/ml, prior to 32 weeks gestation. After 31 weeks gestation up to term, the lactoferrin levels increase markedly in nonlaboring patients. These findings are consistent with the work of Niemela et al.,[12] who measured lactoferrin levels in AF samples from 109 pregnancies at 14–42 weeks and found a striking

TABLE 1. Infected PTL group, <32 weeks

| Gestational age (weeks) | Cx[a] | Organism | Placental histology | Lactoferrin, (μg/ml) |
|---|---|---|---|---|
| 28 | – | | + | 5.361 |
| 29 | + | *Fusobacterium nucleatum* | + | 4.879 |
| 28 | + | *Mycoplasma hominis* | + | 2.754 |
| 28 | – | | + | 0.788 |
| 29 | – | | + | 5.978 |
| 29 | – | | + | 2.589 |
| 24 | + | *Gardnerella vaginalis* *F. nucleatum* | + | 9.017 |
| 27 | + | *M. hominis* *F. nucleatum* | + | 9.341 |
| 26 | + | *Candida albicans* | + | 10.386 |
| 30 | – | | + | 0.907 |
| 29 | – | | + | 10.370 |

[a]Cx = AF culture results.

increase in AF lactoferrin levels in the third trimester. These workers also measured lactoferrin in numerous fetomaternal tissues at term and found the highest levels of lactoferrin were present in decidua compared with amnion, chorion, trophoblast, umbilical cord, and fetal serum.

These findings suggest a role for lactoferrin in the normal immune mechanisms of pregnancy. It is known that lactoferrin plays a protective role by interfering with the bacterial utilization of iron molecules crucial for cell growth and metabolism.[16,17] Lactoferrin also has bacteriostatic and bacteriocidal effects in a wide range of organisms, including gram-positive and gram-negative bacteria, aerobes, anaerobes, and yeasts.[18] The iron-lactoferrin complex may also provide iron that is necessary for catalyzing the production of free radicals, resulting in the killing of phagocytized bacteria.[19,20] Further evidence supporting the role of lactoferrin in the inflammatory reaction comes from the clinical arena where it is known that patients lacking specific granules in their neutrophils have recurrent bacterial infections.[21] These data suggest that lactoferrin plays a protective role against invading pathogens in the AF of late pregnancy. We also postulate that the source of the increasing AF lactoferrin in the third trimester of normal pregnancy is the decidua and that rising lactoferrin levels after 31 weeks gestation are a marker for decidual activation in possible preparation for term labor. Lactoferrin levels may rise after 31 weeks in preparation for the inevitable invasion of pathogens which will accompany the normal labor process.

Our data also show that AF lactoferrin levels are markedly elevated in PTL patients with IAI at <32 weeks gestation. This finding was not related to increasing gestational age. We propose that the source of lactoferrin in these patients is the secondary granules of activated neutrophils, and the finding of elevated AF lactoferrin at <32 weeks gestation is a marker for IAI. Since lactoferrin levels are normally quite low at these gestational ages, we propose that these patients are at increased risk for IAI because they lack the protective effect of decidual lactoferrin which normally increases at about 32 weeks.

Using a lactoferrin level of >2.5 μg/ml, we were able to identify all of the patients at <32 weeks with positive AF cultures. We propose that an AF lactoferrin higher than this critical value at gestations of <32 weeks is highly suggestive of IAI.

We recognize the limitations of our current technology in identifying those PTL patients without clinical evidence of chorioamnionitis who actually have an infectious etiology for their PTL. We used the most widely reported methods in the literature for identifying IAI: AF culture and placental histologic chorioamnionitis. The AF culture is positive in a minority (0–26%) of PTL patients with intact membranes[10] and may not reflect infectious events occurring in the decidua or membranes. The placental histology at delivery may not be an accurate reflection of the events which initiated PTL. Despite the limitations of these methods, our data add further to the evidence supporting the hypothesis that PTL has an infectious etiology.

Further study of the role of lactoferrin in normal and abnormal gestation is required. In particular, the source of lactoferrin (decidua vs. neutrophil) in various clinical situations must be determined. The results of this study lend further support to theories suggesting infection as a major cause of PTL, especially in very preterm gestations. The AF lactoferrin assay may prove to be a useful tool to clinicians in selecting those PTL patients at <32 weeks gestation who may benefit from closer observation, early delivery, or antimicrobial therapy.

### REFERENCES

1. Chamberlain G: Epidemiology and aetiology of the preterm baby. Clin Obstet Gynecol 11:297–314, 1984.
2. Bobitt JR, Hayslip CC, Damato JD: Amniotic fluid infection as determined by transabdominal amniocentesis in patients with intact membranes in preterm labor. Am J Obstet Gynecol 140:947, 1981.
3. Wallace RL, Herrick CN: Amniocentesis in the evaluation of preterm labor. Obstet Gynecol 57:483, 1981.
4. Wahbeh CJ, Hill GB, Eden RD, et al.: Intra-amniotic bacterial colonization in preterm labor. Am J Obstet Gynecol 148:739, 1984.
5. Weible DR, Randal HW: Evaluation of amniotic fluid in preterm labor with intact membranes. J Reprod Med 30:777, 1985.
6. Leigh J, Garite TJ: Amniocentesis and the management of premature labor. Obstet Gynecol 67:500, 1986.
7. Duff P, Kopelman JN: Subclinical intraamniotic infection with asymptomatic patients with refractory preterm labor. Obstet Gynecol 69:756, 1987.
8. Hillier SL, Witkin SS, Krohn MA, Watts DH, Kiviat WB, Eschenback DA: The relationship of amniotic fluid cytokines and preterm delivery, amniotic fluid infection, histologic chorioamnionitis, and chorioamnion infection. Obstet Gynecol 81:941–948, 1993.
9. Pankuch GA, Appelbaum PC, Lorenz RP, et al.: Placental microbiology and histology and the pathogenesis of chorioamnionitis. Obstet Gynecol 64:802, 1984.
10. Romero R, Sirtori M, Oyarzun E, Avila C, Mazor M, Callahan R, Sabor V, Athanassiadis AP, Hobbins JC: Infection and labor. V. Prevalence, microbiology, and clinical significance of intraamniotic infection in women with preterm labor and intact membranes. Am J Obstet Gynecol 161:817–824, 1989.
11. Levay PF, Viljoen M: Lactoferrin: A general review. Haematologica 80:252–267, 1995.
12. Niemela A, Kulomaa M, Vilja P, Tuohimaa P, Saarikoski S: Lactoferrin in human amniotic fluid. Hum Reprod 4:99–101, 1989.
13. Greig PC, Ernest JM, Teot L, Erikson M, Talley R: Amniotic fluid IL-6 levels correlate with histologic chorioamnionitis and amniotic fluid cultures in patients in preterm labor with intact membranes. Am J Obstet Gynecol 169:1035–1044, 1993.
14. Salafia CM, Weigl C, Silberman L: The prevalence and distribution of acute placental inflammation in uncomplicated term pregnancy. Obstet Gynecol 73:383–389, 1989.
15. Gibbs RS, Blanco JD, St. Clair PJ, Castaneda YS: Quantitative bacteriology of amniotic fluid from women with clinical intraamniotic infection at term. J Infect Dis 145:1–8, 1982.
16. Sanchez L, Calvo M, Brock JH: Biological role of lactoferrin. Arch Dis Child 67:657, 1992.
17. Iyer S, Lonnerdal B: Lactoferrin, lactoferrin receptors, and iron metabolism. Eur J Clin Nutr 47:232, 1993.
18. Weinberg ED: Iron withholding: A defense against infection and neoplasia. Physiol Rev 64:65, 1984.
19. Lima MF, Kierszenbaum F: Lactoferrin effects on phagocytic cell function. II. The presence of iron is required for the lactoferrin molecule to stimulate intracellular killing by macrophages but not to enhance uptake of particles and microorganisms. J Immunol 139: 1647, 1987.
20. Aruoma OI, Halliwell B: Superoxide-dependent and ascorbate-dependent formation of hydroxyl radicals from hydrogen peroxide in the presence of iron. Are lactoferrin and transferrin promoters of hydroxyl-radical generation? Biochem J 241:273, 1987.
21. Breton-Gorius J, Mason DY, Buriot D, Vilde JL, Griscelli C: Lactoferrin deficiency as a consequence of a lack of specific granules in neutrophils from a patient with recurrent infections. Am J Pathol 99:413, 1980.
